# Supplementary material for: Integrated cross-study datasets of genetic dependencies in cancer
Source: Nat Commun. 2021 Mar 12;12:1661. doi: 10.1038/s41467-021-21898-7 (PMC7955067; doi:10.1038/s41467-021-21898-7)
Supplement: Supplementary file 1 — Supplementary Information [file 41467_2021_21898_MOESM1_ESM.pdf]

Supplementary Information for

Integrated cross-study datasets of genetic dependencies in cancer

Pacini et al.

Supplementary Figures

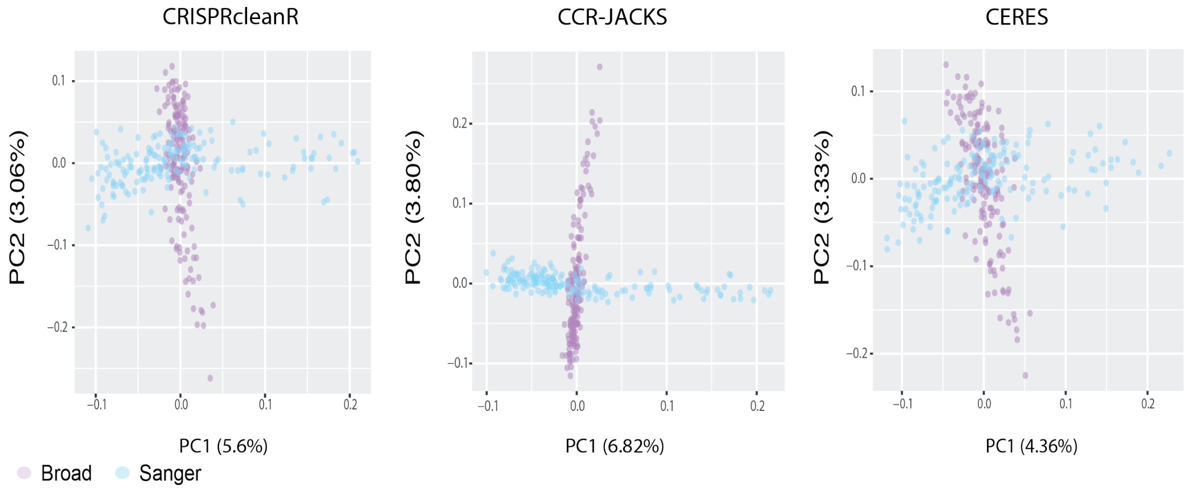

Supplementary Figure 1: Residual batch effects following correction across pre-processing methods. Principal component plots of the gene dependency profiles of cell lines screened in both Broad and Sanger studies (168 cell lines) following ComBat batch correction, across different pre-processing methods. The colours indicate the origin of the screen, with Broad screens in purple and Sanger screens in blue.

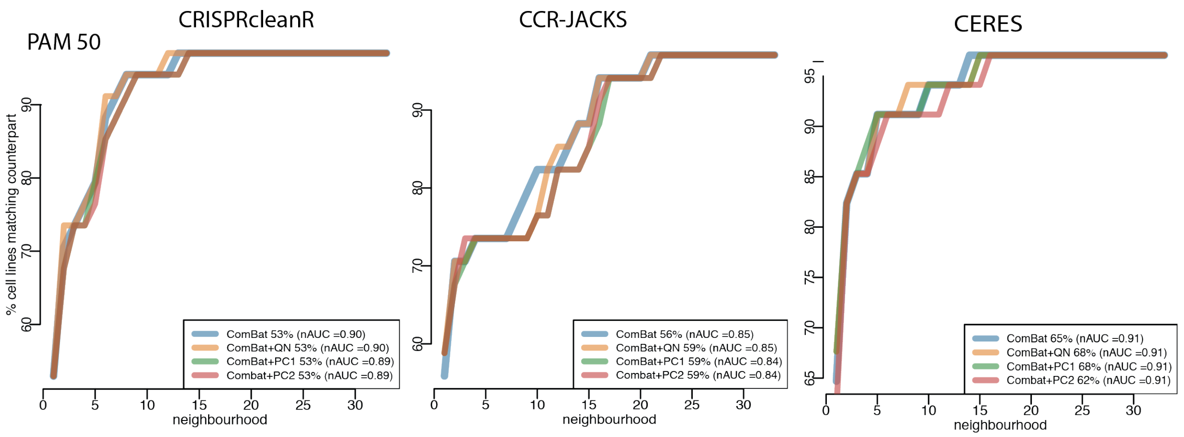

Supplementary Figure 2: Lineage subtype identification.

Agreement of Breast CRISPR-cas9 fitness profiles according to the clinical Breast PAM50 cancer subtypes. For each query Breast cancer cell line in turn we computed correlation scores to all other Breast cancer cell lines (responses). We then ranked the response cell lines according to these correlations. For each query cell line, the rank position k of the most correlated response cell line from the same cancer subtype (matching response) was identified. A rank of k = 1 indicates that the query cell line was closest to another cell line from the same cancer subtype. The curves show the ratio of query cell lines with a matching response with a given rank position. The proportion of query cell lines with a matching response in k = 1 are also shown as percentages for each dataset. The normalised area under the curve (nAUC) for each dataset is shown in brackets.

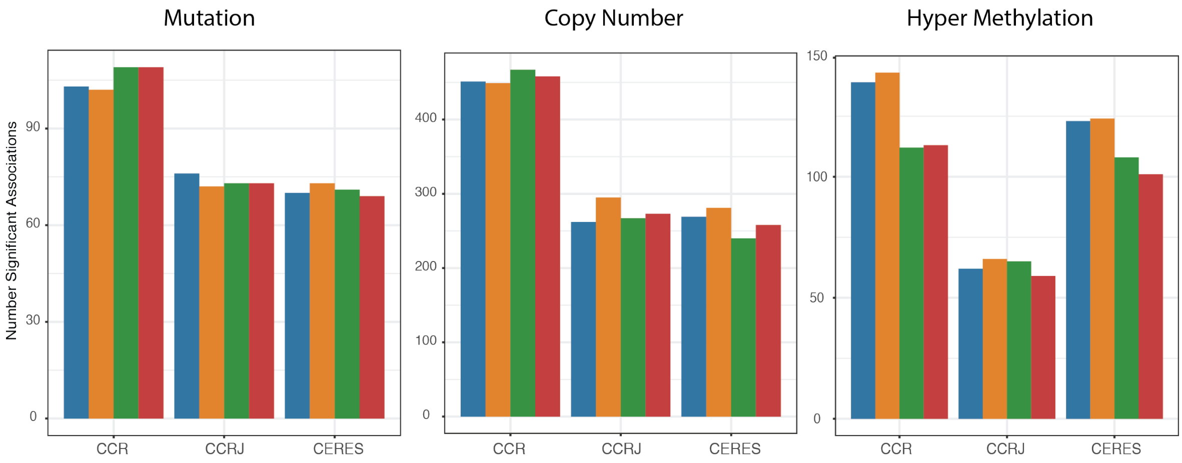

Supplementary Figure 3: Identification of Biomarker/Dependency associations

Cancer functional-events/dependency associations found for each biomarker type and dataset at 5% FDR. Associations were tested using a two-sided t-test and multiple hypothesis corrected using Benjamini-Hochberg. The three pre-processing methods are shown CRISPRcleanR (CCR), CRISPRcleanR with JACKS (CCRJ) and CERES. The colour of the bars indicates the batch correction method used. ComBat only is shown in blue, ComBat with quantile normalisation in orange. The first principal component removed from the ComBat with quantile normalisation method is in green and with the first two principal components removed in red.

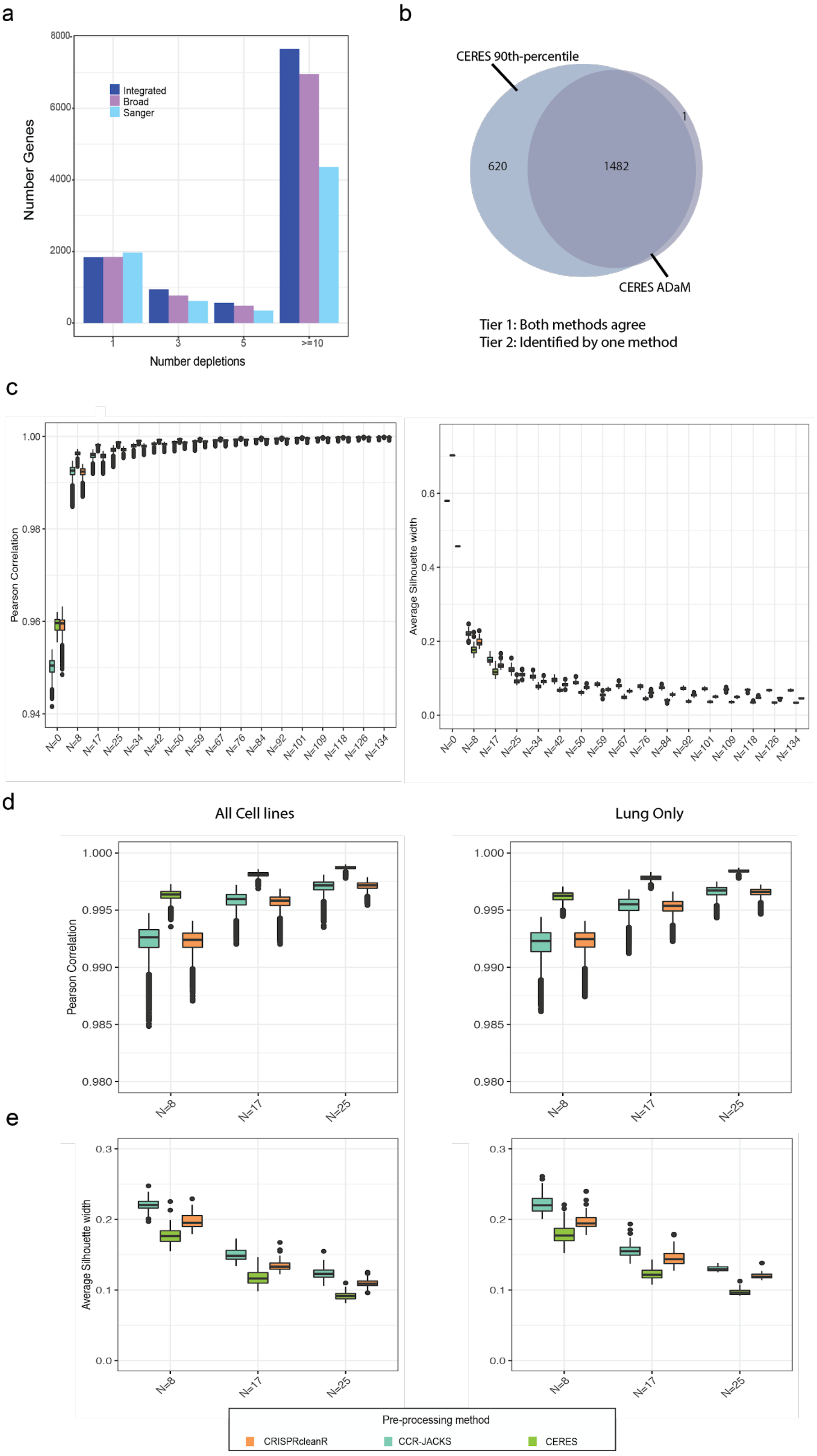
a
b
Number Genes
8000
6000
4000
2000
0
Integrated
Broad
Sanger
1
3
5
>=10
Number depletions
CERES 90th-percentile
620
1482
1
CERES ADaM
Tier 1: Both methods agree
Tier 2: Identified by one method
c
Pearson Correlation
1.00
0.98
0.96
0.94
N=0
N=8
N=17
N=25
N=34
N=42
N=50
N=59
N=67
N=76
N=84
N=92
N=101
N=109
N=118
N=126
N=134
Average Silhouette width
0.6
0.4
0.2
0.0
d
All Cell lines
Lung Only
Pearson Correlation
1.000
0.995
0.990
0.985
0.980
N=8
N=17
N=25
e
Average Silhouette width
0.3
0.2
0.1
0.0
Pre-processing method
CRISPRcleanR
CCR-JACKS
CERES

Supplementary Figure 4: Performance of the Integrated Dataset. a. Number of genes that are significant dependencies (at 5% FDR) in fixed numbers of cell lines across individual datasets and the integrated one, for CRISPRcleanR pre-processing methods. b. Venn diagram showing genes called common essential when using two different detection algorithms (ADaM and 90th-percentile) applied to the CERES processed dataset. c. The boxplots contain *N*=50 random samples of between 5% and 90% of the 168 overlapping cell lines (number of cell lines in each sample indicated on the x-axis). The boxplot shows the interquartile range in the box with the median as a horizontal line. Whiskers extend to 1.5 times the interquartile range and outlier points outside this range plotted. For each sample the Pearson correlation of the DPGs following ComBat correction compared to the integrated dataset was calculated for each pre-processing method. We also include *N*=0 as the correlation between the dataset with no batch correction applied. In the right hand plot the average silhouette width (ASW) for each down sampled dataset was calculated using the institute of origin as the cluster label. An ASW of close to zero indicating a near random performance of the clustering, meaning the samples do not cluster by the origin of the screen and batch effects have been removed. d. The boxplots contain *N*=50 random samples of 8, 17 or 25 cell lines from the overlapping set of 168 cell lines on the left plot. The right-hand plot shows the results when using 8, 17 or 25 cell lines drawn only from the set of Lung cancer cell lines. The box shows the interquartile range with the median drawn as a horizontal line. Whiskers extend to 1.5 times the interquartile range and outlier points outside this range plotted. For each sample the Pearson correlation of the DPGs following ComBat correction compared to the integrated dataset was calculated for each pre-processing method. e. The average silhouette width (ASW) for each down sampled dataset was calculated using the institute of origin as the cluster label. An ASW of close to zero indicating a near random performance of the clustering, meaning the samples do not cluster by the origin of the screen and batch effects have been removed. The left-hand plot draws random samples of cell lines from all lineages, the right-hand plot uses Lung cancer cell lines only. The boxplot shows the interquartile range in the box with the median as a horizontal line. Whiskers extend to 1.5 times the interquartile range and outlier points outside this range plotted.

Supplementary Tables

Supplementary Table 1 - Top 20 list of gene ontology Biological Process gene sets from MsigDB unique to the common essential gene set of the integrated data. Tests were performed using a hypergeometric test and p-values were multiple hypothesis corrected using Benjamini-Hochberg (FDR q-value).

Gene Set Name	# Genes in Gene Se	# Genes in Overlap	k/K	p-value	FDR q-value
GO_PROTEIN_CONTAINING_COMPLEX_SUBUNIT_ORGANIZATION	1978	44	0.0222	1.56E-14	1.18E-10
GO_APOPTOTIC_PROCESS	1994	42	0.0211	4.13E-13	1.57E-09
GO_CELL_CYCLE	1881	40	0.0213	1.21E-12	3.06E-09
GO_REGULATION_OF_ORGANELLE_ORGANIZATION	1310	32	0.0244	8.43E-12	1.60E-08
GO_MITOCHONDRIAL_TRANSLATION	135	12	0.0889	2.77E-11	4.19E-08
GO_MITOCHONDRIAL_GENE_EXPRESSION	165	12	0.0727	2.90E-10	3.66E-07
GO_ORGANONITROGEN_COMPOUND_BIOSYNTHETIC_PROCESS	1827	35	0.0192	6.05E-10	6.20E-07
GO_REGULATION_OF_CELL_DEATH	1746	34	0.0195	7.23E-10	6.20E-07
GO_AMIDE_BIOSYNTHETIC_PROCESS	904	24	0.0265	8.10E-10	6.20E-07
GO_PEPTIDE_METABOLIC_PROCESS	907	24	0.0265	8.64E-10	6.20E-07
GO_PEPTIDE_BIOSYNTHETIC_PROCESS	761	22	0.0289	9.01E-10	6.20E-07
GO_CELL_CYCLE_PROCESS	1422	30	0.0211	1.29E-09	8.15E-07
GO_CELLULAR_AMIDE_METABOLIC_PROCESS	1192	27	0.0227	2.08E-09	1.21E-06
GO_CELLULAR_PROTEIN_CONTAINING_COMPLEX_ASSEMBLY	1129	26	0.023	3.01E-09	1.63E-06
GO_TRANSLATIONAL_ELONGATION	135	10	0.0741	7.60E-09	3.84E-06
GO_MITOTIC_CELL_CYCLE	1053	24	0.0228	1.54E-08	7.30E-06
GO_CELLULAR_MACROMOLECULE_CATABOLIC_PROCESS	1201	25	0.0208	4.42E-08	1.97E-05
GO_POSITIVE_REGULATION_OF_ORGANELLE_ORGANIZATION	638	18	0.0282	4.67E-08	1.97E-05
GO_MITOCHONDRIAL_TRANSLATIONAL_TERMINATION	89	8	0.0899	5.46E-08	2.18E-05
GO_MACROMOLECULE_CATABOLIC_PROCESS	1431	27	0.0189	9.03E-08	3.42E-05

Biomarkers found in the integrated dataset but not in the Sanger individual one								
**CFE**	**GENE**	**delta**	**effect size**	**p-value**	**no. positive**	**no. negative**	**tissue**	**FDR**
MLL2_mut	WRN	-2.312	3.686	1.18E-07	4	28	Ovary	0.002754
LRPPRC_mut	DMXL1	-0.757	3.866	2.53E-07	3	33	Large Intestine	0.005490
TP53_mut	MDM2	1.280	2.304	4.87E-07	26	10	Large Intestine	0.007412
NRAS_mut	NRAS	-3.000	5.918	5.96E-22	10	41	Haematopoietic and Lymphoid	0.000000
TP53_mut	MDM2	0.691	1.445	5.87E-06	30	21	Haematopoietic and Lymphoid	0.009438
G.1q21.3.	IRS1	-0.519	2.939	8.27E-07	4	47	Haematopoietic and Lymphoid	0.001899
G.1q22.	IRS1	-0.519	2.939	8.27E-07	4	47	Haematopoietic and Lymphoid	0.001899
G.1q23.2..SDHC.	IRS1	-0.519	2.939	8.27E-07	4	47	Haematopoietic and Lymphoid	0.001899
L.1p32.3..CDKN2C.FAF1.	MAF	-1.266	2.746	4.24E-07	5	46	Haematopoietic and Lymphoid	0.001311
L.1p32.3..CDKN2C.FAF1.	SMAD7	-1.825	2.883	1.52E-07	5	46	Haematopoietic and Lymphoid	0.000764
chr1.181451311.181452049.._HypMET	ZEB1	-0.439	1.670	4.58E-07	21	30	Haematopoietic and Lymphoid	0.001363
chr3.139258152.139258731.RBP1._HypMET	ZEB1	-0.422	1.658	6.16E-07	20	31	Haematopoietic and Lymphoid	0.001707
chr3.99594969.99595215.C3orf26..FILIP1L..MIR548G._HypMET	ZEB1	-0.420	1.582	5.22E-06	15	36	Haematopoietic and Lymphoid	0.008736
EGFR_mut	TMEM170B	-0.049	2.502	4.23E-06	4	89	Lung	0.008740
INPPL1_mut	MET	-1.276	3.365	1.27E-07	3	90	Lung	0.000878
KRAS_mut	KRAS	-1.659	2.587	1.59E-18	25	68	Lung	0.000000
MLL2_mut	THAP3	-0.301	1.979	2.40E-06	7	86	Lung	0.005630
NRAS_mut	NRAS	-1.468	6.738	8.00E-23	4	89	Lung	0.000000
SMARCA4_mut	SMARCA2	-0.703	1.272	3.48E-06	19	74	Lung	0.007505
TP53_mut	MDM2	0.782	1.848	5.13E-11	72	21	Lung	0.000002
TP53_mut	MDM4	0.549	1.236	2.96E-06	72	21	Lung	0.006544
TP53_mut	PPM1D	0.177	1.291	1.20E-06	72	21	Lung	0.003600
TP53_mut	TP53	-0.726	1.753	3.09E-10	72	21	Lung	0.000007
TP53_mut	USP28	-0.444	1.223	3.63E-06	72	21	Lung	0.007700
L.19p13.3..GNA11.	ZSWIM7	-0.432	2.369	1.48E-06	5	88	Lung	0.004110
L.19p13.3.	ZSWIM7	-0.432	2.369	1.48E-06	5	88	Lung	0.004110
G.10p12.1..CUL2.EPC1.	FBXO42	-0.835	2.013	4.39E-07	8	85	Lung	0.001793
G.8q23.3..RAD21.	SATB2	-0.147	1.906	4.55E-07	9	84	Lung	0.001820
G.9q21.11..GNAQ.NTRK2.PCSK5.TJP2.	FAM83D	-0.693	2.980	2.01E-06	3	90	Lung	0.005135
G.9q21.11..GNAQ.NTRK2.PCSK5.TJP2.	MET	-1.261	3.234	3.30E-07	3	90	Lung	0.001572
G.12q13.3..CDK4.	HMGA2	-0.044	2.366	1.52E-06	5	88	Lung	0.004179
G.3q27.1.	SLC25A28	-0.553	2.750	5.75E-07	4	89	Lung	0.002084
G.1q21.2..PIP5K1A.SETDB1.	NUP37	-0.263	2.048	2.94E-07	8	85	Lung	0.001569
G.1q21.2..PIP5K1A.SETDB1.	TTC7A	-0.845	1.797	4.89E-06	8	85	Lung	0.009457
G.1q25.3.	C11orf53	-0.350	2.590	2.11E-06	4	89	Lung	0.005135
G.1q25.3.	MYB	-0.319	2.940	1.17E-07	4	89	Lung	0.000865
G.1q25.3..DHX9.	ELF3	-0.904	3.174	5.10E-07	3	90	Lung	0.001962
G.1q32.2.	ELF3	-0.904	3.174	5.10E-07	3	90	Lung	0.001962
G.17q12..CDK12.ERBB2.MED24.	ELF3	-0.941	2.478	5.10E-06	4	89	Lung	0.009523
L.17p13.2..2	MAGEH1	-0.015	2.532	3.34E-06	4	89	Lung	0.007290

G.14q21.3..GNG2.SOS2.	ERBB2	-1.252	2.480	5.01E-06	4	89	Lung	0.009457
chr12.104697348.104697984.EID3..TXNRD1._HypMET	SNAI2	-0.421	2.560	2.66E-06	4	89	Lung	0.006021
chr4.102711829.102712199.BANK1._HypMET	TP63	-0.399	1.872	2.34E-07	10	83	Lung	0.001405
chr6.26250436.26250827.HIST1H2BH..HIST1H3F._HypMET	UXS1	-0.599	1.186	2.06E-06	25	68	Lung	0.005135
TP53_mut	TP53	-0.978	1.910	2.28E-07	27	17	Central Nervous System	0.006248
G.16q12.2..MMP2.	UAP1	-1.104	4.590	1.20E-08	3	31	Breast	0.000655
G.17q12..CDK12.ERBB2.MED24.	ERBB2	-2.060	2.716	6.44E-08	9	25	Breast	0.001760
G.17q22..CLTC.PPM1D.	FOXA1	-3.443	3.047	1.18E-07	6	28	Breast	0.002578
G.17q12.	JUP	-2.580	5.104	4.02E-09	3	27	Esophagus	0.000130
G.17q12..CDK12.ERBB2.MED24.	ERBB2	-2.873	3.283	2.77E-08	7	23	Esophagus	0.000597
G.20q13.13.	TCF7L2	-1.019	3.485	4.96E-07	4	26	Esophagus	0.006413
G.20q13.2..ARFGAP1.GNAS.	TCF7L2	-1.019	3.485	4.96E-07	4	26	Esophagus	0.006413

Biomarkers found in the integrated dataset but not in the Broad individual one

CFE	GENE	delta	effect size	p-value	no. positive	no. negative	tissue	FDR
MSI	WRN	-0.802901	3.66484756	4.94E-09	6	26	Ovary	0.00023127
MLL2_mut	WRN	-1.315957	4.82489608	2.66E-14	9	27	Large Intestine	3.14E-09
TP53_mut	TP53	-0.935904	2.57878432	5.48E-08	26	10	Large Intestine	0.00161749
MSI	WRN	-1.463708	4.34947025	8.57E-14	11	25	Large Intestine	5.06E-09
TP53_mut	MDM2	0.777913	1.44545205	5.87E-06	30	21	Haematopoietic and Lymphoid	0.00943769
L.18q22.1.	CCND1	-1.237011	2.96301292	7.59E-07	5	46	Haematopoietic and Lymphoid	0.00189934
L.11q24.2.	MEF2B	-1.285661	3.37202144	7.62E-07	3	48	Haematopoietic and Lymphoid	0.00189934
L.11q24.2..1	MEF2B	-1.285661	3.37202144	7.62E-07	3	48	Haematopoietic and Lymphoid	0.00189934
G.8q24.21..MYC.	RELA	-0.790478	2.50025958	2.65E-06	5	46	Haematopoietic and Lymphoid	0.00484819
L.6q23.1.	FBXO42	-0.771001	3.02422845	5.84E-06	3	48	Haematopoietic and Lymphoid	0.00943769
L.1p32.3..CDKN2C.FAF1.	SMAD7	-1.013554	2.88251952	1.52E-07	5	46	Haematopoietic and Lymphoid	0.00076409
L.1p13.2..ATP1A1.CSDE1.NRAS.	TCF3	-1.288361	2.69716639	4.18E-06	4	47	Haematopoietic and Lymphoid	0.00747206
L.2q32.1.	STAT3	-1.461933	3.44835288	4.85E-07	3	48	Haematopoietic and Lymphoid	0.00139123
chr1.181451311.181452049.._HypMET	ZEB1	-0.453569	1.67009244	4.58E-07	21	30	Haematopoietic and Lymphoid	0.00136252
chr1.248100325.248100726.OR2L13._HypMET	CEBPA	-0.861881	3.4379565	2.27E-09	5	46	Haematopoietic and Lymphoid	2.28E-05
chr15.45315201.45315543.SORD._HypMET	CEBPA	-0.685158	2.44758305	8.60E-07	6	45	Haematopoietic and Lymphoid	0.00192048
chr2.85811340.85811855.VAMP5._HypMET	CEBPA	-0.710293	2.44394707	6.81E-08	8	43	Haematopoietic and Lymphoid	0.00042097

chr3.139258152.139258731.RBP1._HypMET	ZEB1	-0.453569	1.65809762	6.16E-07	20	31	Haematopoietic and Lymphoid	0.00170702
chr3.99594969.99595215.C3orf26..FILIP1L..MIR548G._HypMET	ZEB1	-0.466651	1.58234806	5.22E-06	15	36	Haematopoietic and Lymphoid	0.00873631
chr5.67584213.67584451.PIK3R1._HypMET	CEBPA	-0.710293	2.44394707	6.81E-08	8	43	Haematopoietic and Lymphoid	0.00042097
chr7.143582125.143582610.FAM115A._HypMET	CEBPA	-0.621492	2.1507397	1.01E-06	8	43	Haematopoietic and Lymphoid	0.00219552
chr7.143582125.143582610.FAM115A._HypMET	SPI1	-1.250823	2.07147242	2.26E-06	8	43	Haematopoietic and Lymphoid	0.00423081
chr9.140310894.140312457.EXD3._HypMET	CEBPA	-0.739537	2.77681828	3.12E-09	8	43	Haematopoietic and Lymphoid	2.79E-05
chr9.140310894.140312457.EXD3._HypMET	GFI1	-0.709274	2.07171004	2.26E-06	8	43	Haematopoietic and Lymphoid	0.00423081
FAT1_mut	LRP8	-0.664691	2.81774308	3.27E-07	4	89	Lung	0.00157233
MLL2_mut	THAP3	-0.327097	1.97895289	2.40E-06	7	86	Lung	0.00563011
NF2_mut	LMNA	-0.681437	2.24732201	4.35E-06	5	88	Lung	0.00880334
TP53_mut	MDM4	0.296693	1.23592817	2.96E-06	72	21	Lung	0.00654374
TP53_mut	USP28	-0.238877	1.22349045	3.63E-06	72	21	Lung	0.00770018
ZNF292_mut	MAGEH1	-0.85731	2.98369601	1.97E-06	3	90	Lung	0.00510659
L.19p13.3..GNA11.	ZSWIM7	-0.688176	2.36937224	1.48E-06	5	88	Lung	0.00410954
L.19p13.3.	ZSWIM7	-0.688176	2.36937224	1.48E-06	5	88	Lung	0.00410954
L.10p13.	ISG15	-0.386527	2.07071545	9.17E-07	7	86	Lung	0.00298617
L.10p13..1	ISG15	-0.386527	2.07071545	9.17E-07	7	86	Lung	0.00298617
L.10p12.2.	ISG15	-0.47717	2.52615932	4.23E-08	6	87	Lung	0.0004514
L.10q21.1.	MET	-0.117277	2.34503926	1.83E-06	5	88	Lung	0.00483005
L.10q21.1.	VDAC2	-0.692858	2.92835025	7.61E-09	5	88	Lung	0.00011249
G.8q23.3..RAD21.	SATB2	-0.392371	1.90637869	4.55E-07	9	84	Lung	0.00181983
G.16q12.2..MMP2.	ARHGDIA	-0.789897	2.45455983	6.82E-07	5	88	Lung	0.00238404
L.16p13.3.	COLCA2	-0.76439	3.03220125	1.40E-06	3	90	Lung	0.00401372
L.16p13.3.	MAGEH1	-0.877768	3.36370347	1.28E-07	3	90	Lung	0.00087836
L.16p13.3.	SLC39A6	-0.914813	4.03026822	7.86E-10	3	90	Lung	1.51E-05
L.16p13.3..2	COLCA2	-0.781345	3.30799257	1.93E-07	3	90	Lung	0.00119499
L.16q22.2..ZFHX3.	COLCA2	-0.692093	2.69322487	9.13E-07	4	89	Lung	0.00298617
L.9q33.2..PTGS1.	AK2	-0.686416	2.84883592	4.99E-06	3	90	Lung	0.00945651
L.9q33.2..PTGS1.	MAGEH1	-0.776974	3.0669125	1.10E-06	3	90	Lung	0.00335591
L.9q33.3.	AK2	-0.686416	2.84883592	4.99E-06	3	90	Lung	0.00945651
L.9q33.3.	MAGEH1	-0.776974	3.0669125	1.10E-06	3	90	Lung	0.00335591
G.3p14.1..FOXP1.MITF.	SQSTM1	-0.460289	2.50205642	4.23E-06	4	89	Lung	0.00873951
L.3p21.2.	CCND2	-0.827033	1.89768735	1.62E-06	8	85	Lung	0.00432272
G.1q21.2..PIP5K1A.SETDB1.	TTC7A	-0.255027	1.79703465	4.89E-06	8	85	Lung	0.00945651

L.17p13.1.	RAB21	-0.906042	2.69292284	9.15E-07	4	89	Lung	0.00298617
L.17p13.1..1	SLC33A1	-0.841853	2.23097311	5.02E-06	5	88	Lung	0.00945651
G.14q21.3..GNG2.SOS2.	ERBB2	-0.597859	2.48041538	5.01E-06	4	89	Lung	0.00945651
chr12.104697348.104697984.EID3..TXNRD1._HypMET	SNAI2	-0.558487	2.56047242	2.66E-06	4	89	Lung	0.00602149
chr21.47602431.47602740.C21orf56._HypMET	NFIB	-0.82167	2.33012179	2.10E-06	5	88	Lung	0.00513517
chr6.26250436.26250827.HIST1H2BH..HIST1H3F._HypMET	UXS1	-0.698137	1.18649369	2.06E-06	25	68	Lung	0.00513517
chr8.67344497.67344989.RRS1._HypMET	POU2F3	-0.602781	1.44792804	2.85E-06	14	79	Lung	0.00636087
chr8.67344497.67344989.RRS1._HypMET	SLC30A9	-0.439079	1.46235845	2.32E-06	14	79	Lung	0.00551436
STAG2_mut	STAG1	-0.970975	4.29922162	2.02E-11	5	39	Central Nervous System	1.26E-06
TP53_mut	TP53	-1.071494	1.90962065	2.28E-07	27	17	Central Nervous System	0.0062476
chr1.180881316.180882592.KIAA1614._HypMET	LIFR	-0.655378	3.67128945	2.51E-07	3	41	Central Nervous System	0.0062476
PIK3CA_mut	PIK3CA	-1.014639	3.70125518	1.06E-11	12	22	Breast	1.16E-06
L.3p21.1..CACNA1D.WNT5A.	ITPRIPL1	0.60972	3.716032	7.12E-07	3	31	Breast	0.00972655
L.1p36.32..RPL22.	GTSE1	-0.804368	3.87191854	3.39E-07	3	31	Breast	0.00617567
G.17q12..CDK12.ERBB2.MED24.	ERBB2	-0.925097	2.71622673	6.44E-08	9	25	Breast	0.00176029
G.17q22..CLTC.PPM1D.	FOXA1	-2.531075	3.04723412	1.18E-07	6	28	Breast	0.00257811
G.17q12..CDK12.ERBB2.MED24.	ERBB2	-1.11359	3.28277775	2.77E-08	7	23	Esophagus	0.00059714

Supplementary Table 2 - Lists of tissue specific biomarker/dependency associations found as significant in the integrated dataset but not the individual datasets. Associations were tested using a two-sided *t*-test assuming equal variance and multiple hypothesis corrected using Benjamini-Hochberg. Descriptions of the column headers are given below.

**CFE**	Cancer Functional Event. Potential biomarkers comprising driver mutations, copy number and hypermethylated gene promoters frequently observed in patient tumours
**GENE**	A gene targeted with CRISPR-Cas9
**delta**	Difference in means of the gene fitness effect, splitting cell lines into two groups based on absence/presence of the CFE
**effect size**	Difference in means divided by the pooled standard deviation
**p-value**	P-value indicating significance of difference of the gene fitness effect between the two groups
**no. positive**	Number of cell lines with the CFE
**no. negative**	Number of cell lines without the CFE
**tissue**	Tissue of origin for the cell lines tested
**FDR**	False discovery rate – Benjamini Hochberg adjusted p-value for the test of association between CFE and dependency
