# Supplementary material for: Integrated cross-study datasets of genetic dependencies in cancer
Source: Nat Commun. 2021 Mar 12;12:1661. doi: 10.1038/s41467-021-21898-7 (PMC7955067; doi:10.1038/s41467-021-21898-7)
Supplement: Supplementary file 3 — Description of Additional Supplementary Files [file 41467_2021_21898_MOESM3_ESM.pdf]

Description of Additional Supplementary Files

File Name: Supplementary Data 1
Description: List of cell lines included in at least one of the two individual screens with cross-institute identifiers, lineage and cancer subtype annotations and dataset of origin.

File Name: Supplementary Data 2
Description: Top 10 list of enriched MsigDB gene sets found for the first two principal components of the CRISPRcleanR processed dataset.

File Name: Supplementary Data 3
Description: List of significant tissue specific biomarker and dependency associations under the three different pre-processing methods and four batch correction pipelines.

File Name: Supplementary Data 4
Description: List of common essential genes with associated Tiers.
